# Draft genomes of meropenem-resistant *Pseudomonads* from the cloacae of turtles in Lake Opinicon, Canada

**DOI:** 10.1128/mra.01203-23

**Published:** 2024-02-20

**Authors:** Asalia Ibrahim, Lauren Bradford, Grégory Bulté, Alex Wong

**Affiliations:** 1 Department of Biology, Carleton University, Ottawa, Ontario, Canada; SUNY College of Environmental Science and Forestry, Syracuse, New York, United States

**Keywords:** carbapenems, antimicrobial resistance, *Pseudomonads*

## Abstract

We report the draft genome sequences of seven meropenem-resistant bacterial isolates from the cloacae of painted and musk turtles at Lake Opinicon, Canada. This study improves our understanding of the spread of meropenem-resistant bacteria in the wild.

## ANNOUNCEMENT

Carbapenems are beta-lactam antibiotics used to treat infections caused by multidrug-resistant Gram-negative bacteria. Carbapenems bind to penicillin-binding proteins, disrupting cell wall synthesis and ultimately resulting in cell death. The primary mechanism of clinical carbapenem resistance is the production of carbapenemases, which hydrolyze the amide bond of the beta-lactam ring (1, 2).

Environmental transmission of antimicrobial resistance (AMR) genes and resistant organisms is an increasing concern (3). As a step toward understanding the distribution of AMR in freshwater systems, cloacal swabs were performed on Musk and Painted turtles from three different locations at Lake Opinicon (44°33′30″N 76°19′44″W, ON, Canada): Fishcamp Bay, Stinkpot Bay, and Brooke’s Bay. Swabs were streaked onto lysogeny broth (LB) agar (10 g/L tryptone, 5 g/L yeast extract, and 0.5 g/L NaCl) supplemented with 4 µg/mL meropenem, and then single colonies were streaked onto Uriselect agar (Bio-Rad) for the selection of Gram-negative bacteria based on the ability to ferment lactose. Single colonies were then restreaked onto LB agar supplemented with meropenem. Single colonies were inoculated into LB and grown overnight in a shaking incubator (125 rpm) at 37°C. Genomic DNA was extracted using the One-4-All Genomic DNA Mini-prep Kit (Bio Basic) as described in the manufacturer’s protocol. Whole-genome sequencing was carried out at the Microbial Genome Sequencing Center (Pittsburgh, PA, USA). Sample libraries were prepared using an Illumina DNA Prep kit and IDT 10 bp UDI indices and sequenced on an Illumina NextSeq 2000 producing 2 × 151 bp reads. Reads were trimmed using Trimmomatic 0.36 with sliding window 4:20 and minimum length 36 [Bolger et al., 2014 (4)], and quality control was performed using Fastqc 0.12.1 [Andrews, 2010 (5)]. Genomes were assembled using Unicycler 0.4.4 (6), and the assembly was assessed using Qualimap 2.2.2a (7). Coverage was assessed by mapping reads to the assembly using bowtie2 v2.4.5 (8) and calculating average depth across all positions with the samtools v1.14 depth function (9). Contigs of <200 bp were removed using the BBMAP v38.18 reformat function (10). Default parameters were used except where otherwise noted. The genomes were submitted to NCBI Prokaryotic Genome Annotation Pipeline v6.6 for annotation (11). Resistance genes were identified using ResFinder v4.1.5 (12), and taxonomic classification was performed by the identification of genomic closest average nucleotide identity (ANI) neighbors using LINbase (13).

All seven isolates were classified as members of the genus *Pseudomonas* (Table 1), with nearest neighbors belonging to unnamed species.

**TABLE 1:** Assembly and genome characteristics for the seven isolates.^*a*^

Isolate	Location	Closest ANI neighbor	ANI neighbor % similarity	GC content (%)	No. of contigs	Contig *N*_50_	Total length (bp)	Number of raw reads
PT6	Fishcamp Bay	*Pseudomonas fluorescens* AU11706	91.4	62.3	53	619,162	6,718,404	2,982,833
MT1	Fishcamp Bay	*Pseudomonas fluorescens* AU11706	91.37	62.3	43	812,339	6,880,211	4,618,451
MT15	Stinkpot Bay	*Pseudomonas* sp. MWU12_3153	91.34	62.3	55	647,030	6,795,780	3,394,872
MT16	Stinkpot Bay	*Pseudomonas* sp. MWU12_3153	91.32	62.2	77	667,054	6,655,658	4,061,292
MT23	Brooks Bay	*Pseudomonas* sp. MWU12-2534b	97.92	63.2	89	345,326	6,966,590	3,989,925
MT19	Stinkpot Bay	*Pseudomonas* sp. MWU 341	95.99	62	125	188,314	5,721,847	3,332,021
MT21	Stinkpot Bay	*Pseudomonas* sp. MWU 341	99.22	62	114	238,284	6,233,820	3,632,238

^*a*^ Each isolate was obtained from a different individual turtle.

No known resistance genes were detected by ResFinder in any of the isolates (Table 1). The genomes varied in size between 5,721,847 bp (MT19) and 6,966,590 bp (MT23) in length, and the GC content varied between 62% and 63.2% (Table 1).

These genomes will support further investigations into the spread of carbapenem-resistant bacteria among wild animals. The absence of known carbapenem resistance genes in these meropenem-resistant isolates suggests novel resistance mechanisms. This study also contributes to our understanding of the distribution of *Pseudomonads* in freshwater lakes and their animal inhabitants.

## Data Availability

The assemblies have been deposited in GenBank under BioProject PRJNA999621, with accession numbers JAUTCF000000000.1 to JAUTCL000000000.1. The versions described in the paper are the first versions JAUTCF000000000.1 to JAUTCL000000000.1. The raw sequence data are available under the accessions SRR26919350 to SRR26919356.

## References

[1] Baldwin CM, Lyseng-Williamson KA, Keam SJ. 2008. Meropenem: a review of its use in the treatment of serious bacterial infections. Drugs 68:803–838. doi:10.2165/00003495-200868060-00006 18416587

[2] Codjoe FS, Donkor ES. 2017. Carbapenem resistance: a review. Med Sci (Basel) 6:1. doi:10.3390/medsci6010001 29267233 PMC5872158

[3] Larsson DGJ, Flach C-F. 2022. Antibiotic resistance in the environment. Nat Rev Microbiol 20:257–269. doi:10.1038/s41579-021-00649-x 34737424 PMC8567979

[4] Bolger AM, Lohse M, Usadel B. 2014. Trimmomatic: a flexible trimmer for illumina sequence data. Bioinformatics 30:2114–2120. doi:10.1093/bioinformatics/btu170 24695404 PMC4103590

[5] Andrews S. 2010. FastQC: a quality control tool for high throughput sequence data. Available from: http://www.bioinformatics.babraham.ac.uk/projects/fastqc

[6] Wick RR, Judd LM, Gorrie CL, Holt KE. 2017. Unicycler: resolving bacterial genome assemblies from short and long sequencing reads. PLoS Comput Biol 13:e1005595. doi:10.1371/journal.pcbi.1005595 28594827 PMC5481147

[7] García-Alcalde F, Okonechnikov K, Carbonell J, Cruz LM, Götz S, Tarazona S, Dopazo J, Meyer TF, Conesa A. 2012. Qualimap: evaluating next-generation sequencing alignment data. Bioinformatics 28:2678–2679. doi:10.1093/bioinformatics/bts503 22914218

[8] Danecek P, Bonfield JK, Liddle J, Marshall J, Ohan V, Pollard MO, Whitwham A, Keane T, McCarthy SA, Davies RM, Li H. 2021. Twelve years of SAMtools and BCFtools. Gigascience 10:giab008. doi:10.1093/gigascience/giab008 33590861 PMC7931819

[9] Langmead B, Salzberg SL. 2012. Fast gapped-read alignment with Bowtie 2. Nat Methods 9:357–359. doi:10.1038/nmeth.1923 22388286 PMC3322381

[10] Bushnell B. 2014. BBMap: a fast, accurate, splice-aware aligner (No. LBNL-7065E) Lawrence Berkeley National Lab.(LBNL), Berkeley, CA (United States)

[11] Tatusova T, DiCuccio M, Badretdin A, Chetvernin V, Nawrocki EP, Zaslavsky L, Lomsadze A, Pruitt KD, Borodovsky M, Ostell J. 2016. NCBI prokaryotic genome annotation pipeline. Nucleic Acids Res 44:6614–6624. doi:10.1093/nar/gkw569 27342282 PMC5001611

[12] Zankari E, Hasman H, Cosentino S, Vestergaard M, Rasmussen S, Lund O, Aarestrup FM, Larsen MV. 2012. Identification of acquired antimicrobial resistance genes. J Antimicrob Chemother 67:2640–2644. doi:10.1093/jac/dks261 22782487 PMC3468078

[13] Tian L, Huang C, Mazloom R, Heath LS, Vinatzer BA. 2020. LINbase: a web server for genome-based identification of prokaryotes as members of crowdsourced taxa. Nucleic Acids Res 48:W529–W537. doi:10.1093/nar/gkaa190 32232369 PMC7319462

